# Wide-angle Spectrally Selective Perfect Absorber by Utilizing Dispersionless Tamm Plasmon Polaritons

**DOI:** 10.1038/srep39418

**Published:** 2016-12-19

**Authors:** Chun-hua Xue, Feng Wu, Hai-tao Jiang, Yunhui Li, Ye-wen Zhang, Hong Chen

**Affiliations:** 1 School of Computer Science & Communication Engineering, Guangxi University of Science and Technology, Liuzhou, Guangxi 545006, China; 2 Key Laboratory of Advanced Micro-structure Materials, MOE, School of Physics Science and Engineering, Tongji University, Shanghai 200092, China

## Abstract

We theoretically investigate wide-angle spectrally selective absorber by utilizing dispersionless Tamm plasmon polaritons (TPPs) under TM polarization. TPPs are resonant tunneling effects occurring on the interface between one-dimensional photonic crystals (1DPCs) and metal slab, and their dispersion properties are essentially determined by that of 1DPCs. Our investigations show that dispersionless TPPs can be excited in 1DPCs containing hyperbolic metamaterials (HMMs) on metal substrate. Based on dispersionless TPPs, electromagnetic waves penetrate into metal substrate and are absorbed entirely by lossy metal, exhibiting a narrow-band and wide-angle perfect absorption for TM polarization. Our results exhibit nearly perfect absorption with a value over 98% in the angle of incidence region of 0–80 degree.

Metamaterials are artificial microstructures that exhibit unusual optical properties beyond those available in nature. Appropriately designed metamaterials can be used for engineering electromagnetic (EM) space and controlling the propagation of EM waves, which exhibit many novel phenomena and promote many applications including negative refraction^1^, perfect lenses^2^, cloaking devices^3^ and so on. Recently, another important application, spectrally selective perfect absorbers^4^ have received considerable attention due to their importance for developing sensitive detectors for security related applications^5^ as well as narrow-band thermal emitters for thermophotovoltaic applications^6^,^7^,^8^. In addition to narrow-band response, wide-angle response also is important for spectrally selective absorption since angular directivity of absorption essentially broadens the emission spectrum and thus is detrimental for these applications. Metamaterials including metasurfaces possess the abilities of confining EM fields and engineering photonic dispersion, which can be used for realizing wide-angle spectrally selective absorption. Physically speaking, the excitation of dispersionless optical resonance in the structure with blocked transmission (metal screening or the total reflection regime) can lead to wide-angle optical absorption. On the basis of this mechanism, researchers have realized wide-angle optical absorption in nanostructured metal surfaces^9^ and periodically patterned graphene^10^. Other absorptive microstructures for realizing wide-angle optical absorption include nanodisks^11^,^12^, nanorods^13^, grating^14^ as well as other metallic patterns^15^,^16^.

Based on the excitation of dispersionless Tamm plasmon polaritons, in this paper we explore the possibilities of realizing a wide-angle spectrally selective perfect absorber in a *simple* multilayer structure. The multilayer structure what we consider is a one-dimensional photonic crystal (1DPC) containing hyperbolic metamaterials (HMMs) on a metal substrate. It is well known that an EM surface state (so-called Tamm plasmon polariton, TPP) exists on the interface between 1DPC and metal substrate for frequencies within photonic band gap^17^,^18^. At resonance frequency, there is a buildup of energy on the interface which depends on coupling of evanescent waves, giving up to resonant tunneling effect without reflection. By utilizing the unique properties of resonant tunneling effect, TPPs have exhibited a variety of applications such as optical switching and diode^19^,^20^, second and third harmonic generation enhancement^21^,^22^, lasing^23^ as well as single photon emission^24^. Undoubtedly, the properties of TPPs also can be used for narrow-band perfect absorption.

It is noteworthy that conventional TPPs are always dispersive owing to the angle-dependent properties of photonic band gap^17^,^18^. For obtaining angle-insensitive TPPs, an angle-independent band gap is highly desirable. Very recently, our investigations show the existence of a dispersionless band gap in 1DPC containing HMMs^25^. HMMs are highly anisotropic mediums with hyperbolic dispersion^26^,^27^,^28^ in which one of the principal components of their permittivity (permeability) tensors is opposite in sign to the other two principal components. Since it supports propagating modes with very large wave vectors, HMMs bring about a rich variety of new physics and novel applications such as sub-diffraction imaging^29^, sub-wavelength modes^30^, and spontaneous^31^,^32^,^33^,^34^,^35^ and thermal emission engineering^36^,^37^,^38^. Moreover, in near-infrared or visible region, HMMs can be mimicked by a 1D metal-dielectric stack with subwavelength unit cell^39^,^40^,^41^, which provides a convenient method for experimental implementation of HMMs. It should be pointed out that HMMs not only support high-k waves but also possess anomalous wavevector dispersion by contrast with isotropic dielectric. Anomalous wavevector dispersion in HMMs can compensate normal wavevector dispersion in isotropic dielectric. Such effect, so-called *phase variation compensation effect*, can be used for realizing dispersionless gap in 1DPCs containing HMMs^25^. Since dispersion properties of TPPs depend on that of band gap of 1DPC, we can expect that TPP mode appearing on 1DPC-metal interface will be a dispersionless one. As a result, at resonant wavelength, EM wave penetrating into lossy metal slab and is absorbed entirely. Our results exhibit excellent performance of the structure with a nearly perfect wide-angle absorption with values over 98% in infrared region.

## Structure and Parameters

**Figure 1:**
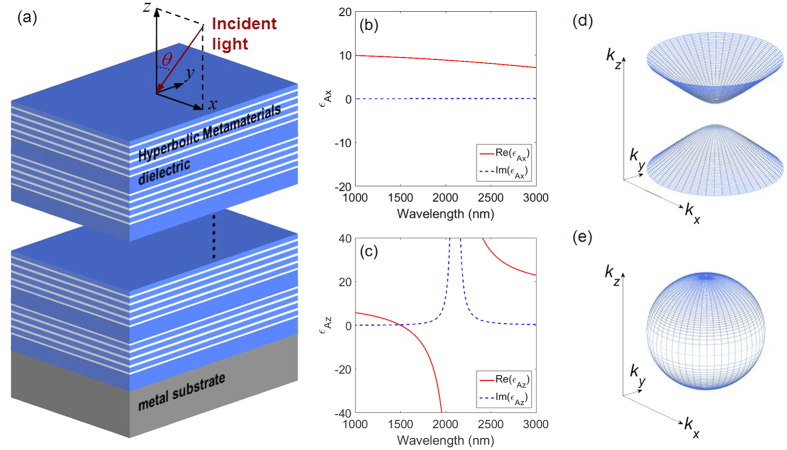
(**a**) The scheme of multilayer structure ((*CD*)_*S*_ *B*)_*N*_ on a metal substrate. Here HMM layer is mimicked by a subwavelength metallodielectric stack (*CD*)_*S*_. TM-polarized EM wave launches into the structure in *x* − *z* plane with angle of incidence *θ*. (**b**) The effective permittivity tensor *ε*_*Ax*_ and (**c**) *ε*_*Az*_ of substructure (*CD*)_*S*_. Layer C is Si with *d*_*C*_ = 39 *nm* and Layer D is ITO with *d*_*D*_ = 11 *nm*. In the wavelength region of 1503~2103 *nm* substructure (*CD*)_*S*_ can be mimicked as type-I HMMs. (**d**) Isofrequency surface in dispersion relation 
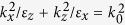
 for type-I HMMs with *ε*_*x*_ > 0 and *ε*_*z*_ < 0, which indicates 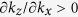. By contrast, isofrequency surface for conventional isotropic medium with *ε*_*x*_ = *ε*_*z*_ > 0 is exhibited in (**e**), which indicates 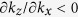.

As is shown in Fig. 1, the multilayer structure proposed is a 1DPC containing HMMs on a metal substrate, which is denoted by ((*CD*)_*S*_ *B*)_*N*_. The structure (*CD*)_*S*_ is a subwavelength metal-dielectric stack with thickness *d*_*c*_ of layer C, thickness *d*_*D*_ of layer D and period number *S*. Thickness *d*_*c*_ + *d*_*D*_ of unit cell is much smaller than wavelength of EM wave within the structure, in the condition of long wavelength limit. Thus the effective-medium approach is valid so that substructure (*CD*)_*S*_ can mimic a HMM layer with thickness *d*_*A*_ = *S(d*_*C*_ + *d*_*D*_) in proper frequency region. Then alternate layers of substructure (*CD*)_*S*_ and dielectric B constitute 1DPC ((*CD*)_*S*_ *B*)_*N*_ with period number *N*. The metal substrate is silver and its permittivity * ε*_*M*_ is referred to ref. 42. The input media are air. Moreover, we consider that electromagnetic waves with transverse-magnetic (TM) polarization launch into the structure in *x* − *z* plane with angle of incidence *θ*. The magnetic field is in the *y* direction.

In the structure, dielectric B and C are selected to be Si with refractive index *n*_*Si*_ = 3.48 (i.e. permittivity 
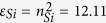
)^42^. Material D is selected to be indium-tin-oxide (ITO). ITO is a good candidate as plasmonic materials in infrared and visible region^43^. The permittivity of ITO is described by a Drude-Lorentz model^44^


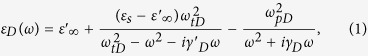


where 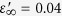 represents the high-frequency dielectric constant, *ε*_*s*_ = 3.48 the strength of Lorentz oscillator, *ω*_*tD*_ = 2*π* × 1640 *THz* the frequency of Lorentz oscillator, 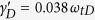 the damping factor of Lorentz oscillator, *ω*_*pD*_ = 2*π* × 375 *THz* the plasma frequency, and *γ*_*D*_ = 0.00645 *ω*_*pD*_ the damping constant.

With respect to structural parameters, we consider *d*_*C*_ = 39 *nm, d*_*D*_ = 11 *nm, d*_*B*_ = 66 *nm, S* = 5 and *N* = 13. For substructure (*CD*)_*S*_, its thickness of unit cell is *d*_*C*_ + *d*_*D*_ = 50 *nm*, which exhibits subwavelength scale in the infrared range, and thus effective-medium approach is available for substructure (*CD*)_*S*_. According to effective-medium approach, substructure (*CD*)_*S*_ can be equivalent to a homogeneous medium A and its components of effective permittivity tensor for TM polarization are given by^27^,^28^


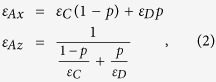


where filling ratio *p* = *d*_*D*_/(*d*_*C*_ + *d*_*D*_) is the volume percentage of layer D in unit cell of substructure. The effective permittivity tensors of substructure (*CD*)_*S*_ are shown in Fig. 1(b) and (c). It can be seen that the effective permittivity tensors Re(*ε*_*Ax*_) > 0 and Re(*ε*_*Az*_) < 0 in the wavelength region of 1503~2103 *nm*. which corresponds to type-I HMMs.

The anomalous wavevector dispersion of HMM provides a specific method for engineering the dispersion of photonic band gap. Figure 1(d) and (e) exhibit isofrequency surfaces of type-I HMMs and isotropic dielectric, respectively. Isofrequency surface of type-I HMMs and isotropic dielectric are given by 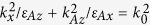 and 
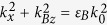
, respectively, where *k*_*Az*_ and *k*_*Bz*_ are the *z* components of wavevector of substructure (*CD*)_*S*_ and dielectric material B, respectively. *k*_*x*_ = *k*_0_ *sin* *θ* is the *x* component of wavevector, and *k*_0_ = *ω/c* is wavevector in vacuum with *ω* being angular frequency and *c* being the speed of light. We can find that 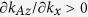 for type-I HMMs and 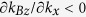 for conventional dielectric material. It means that *k*_*Az*_ will increase with the increase of *θ* but *k*_*Bz*_ will decrease with the increase of *θ*. As a result, HMMs exhibit anomalous wavevector dispersion by contrast with isotropic dielectric. Therefore, when alternate substructure (*CD*)_*S*_ and dielectric layer B constitutes 1DPC ((*CD*)_*S*_ *B*)_*N*_, the increase of *k*_*Az*_ in substructure (*CD*)_*S*_ can compensate the decrease of *k*_*Bz*_ in dielectric layer *B*. Such effect, so-called *phase variation compensation effect*, can be evolved as follows^25^


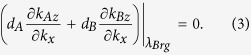


Equation (3) can be used for designing the parameters of 1DPC ((*CD*)_*S*_ *B*)_*N*_ with dispersionless gap. In fact, for the parameters of 1DPC ((*CD*)_*S*_ *B*)_*N*_ in this work, a dispersionless gap with angle-independent Bragg wavelength *λ*_*Brg*_ can be realized on the basis of phase variation compensation effect. In context, we will illustrate its reflection properties in detail.

## Results and Discussion

In the heterostructure composed of 1DPC and metal substrate, it is known that TPPs can exist on 1DPC-metal interface^17^,^18^. For obtaining phase matching condition of TPPs, we assume there is a virtual air layer with thickness *d*_0_ located between 1DPC and metal substrate. Then we use 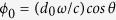 to denote the phase shift of a propagating wave in the virtual air layer, and *ϕ*_*M*_ and *ϕ*_*PC*_ to denote the reflection phases of a propagating wave along z axis upon the metal from the virtual air layer and that upon the 1DPC from the virtual air layer, respectively. With these definitions, the resonance condition of eigenmode of the structure can be written as^17^,^18^


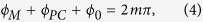


where *m* is zero or an integer. If the length of the virtual air layer *d*_0_ is set to zero and the values of *ϕ*_*M*_ and *ϕ*_*PC*_ are restricted in the interval of (−*π, π*), Eq. (4) can be rewritten as the form


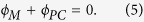


Equation (5) represents phase matching condition of TPPs. The reflection coefficients *r*_*M*_ on metal substrate for a TM-polarized wave is given by the usual Fresnel formula


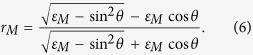


Since in infrared regime the real part of permittivity of metal is the order of −10^2^ and its imaginary part is about 10% of its real part, we assume 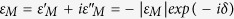 with *δ* being the loss angle. Moreover, we do not consider the case of large angle of incidence so as to avoid cos *θ* → 0. Under these conditions, Eq. (6) can be further derived with the first order Taylor approximation as


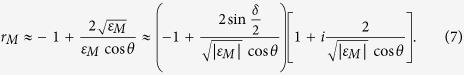


Therefore the reflection phase upon metal substrate yields to


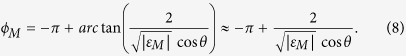


The imaginary part of permittivity of silver will influence the reflection amplitude upon metal substrate but not the reflection phase. Besides, as 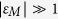, the shift of *ϕ*_*M*_ with respect to optical wavelength is very weak. Return to Eq. (5) and we can find that the dispersion properties of TPPs are essentially determined by that of 1DPCs. It means that we require an angle-insensitive band gap structure to be used for realizing angle-independent TPPs.

**Figure 2:**
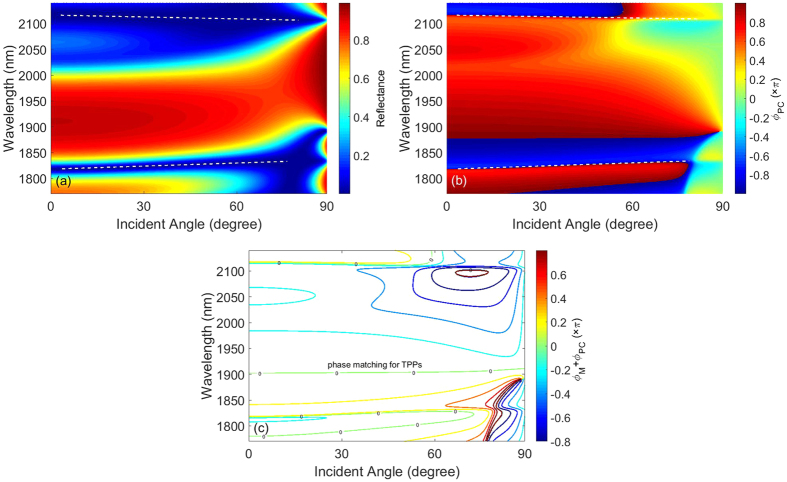
(**a**) The reflectance spectrum and (**b**) reflectance phase upon 1DPC ((*CD*)_*S*_ *B*)_*N*_, respectively. The area surrounded by white dashed line indicates band gap. (**c**) The contour of sum of reflection phases *ϕ*_*M*_ + *ϕ*_*PC*_. Phase matching condition is satisfied at 1902 nm for all angles of incidence.

1DPC containing HMMs provides opportunities for realizing angle-independent TPPs. Using transfer matrix method, the reflectance spectrum and reflection phase shift of a propagating wave along -z axis upon 1DPC ((*CD*)_*S*_ *B*)_*N*_ are given in Fig. 2(a) and (b), respectively. For normal incidence, the reflectance of 1DPC reaches the minimum at 2117 nm and 1818 nm, respectively, which indicates the edges of band gap. Here wavelength 2117 nm corresponds to the lower band edge and 1818 nm corresponds to the upper one. Moreover, for oblique incidence, as is shown by white dashed lines in Fig. 2(a), the shift of band edges is very weak with an increase of angle of incidence. The results show that the shift of lower band edge just is 11 nm and that of upper band edge just is 16 nm, which is much smaller than corresponding optical wavelength. In Fig. 2(b), we also find that the reflection phase *ϕ*_*PC*_ of 1DPC ((*CD*)_*S*_ *B*)_*N*_ shifts from *π* at the lower band edge to −*π* at the upper band edge. It is noteworthy that, in the wavelength region from 1878 nm to 2117 nm, the reflection phase *ϕ*_*PC*_ shifts in the interval of [0, *π*]. Particularly in the vicinity of 1878 nm, *ϕ*_*PC*_ is close to *π*. In such a wavelength, the reflection of 1DPC is analogous with that of magnetic negative materials. It is known that the resonant tunneling effect occurs when 1DPC within band gap exhibits magnetic negative properties^45^. Therefore we can predict that the resonant tunneling effect occurs in the vicinity of 1878 nm. More importantly, for oblique incidence, the shift of *ϕ*_*PC*_ is rather weak with respect to angle of incidence in such a wavelength. Consequently, the resonant tunneling effect occurring in the vicinity of 1878 nm will be insensitive to angle of incidence. In fact, as is shown in Fig. 2(c), the sum of *ϕ*_*M*_ + *ϕ*_*PC*_ is zero in the vicinity of 1902 nm for all angles of incidence, which exhibits wide-angle phase matching for TPP mode.

**Figure 3:**
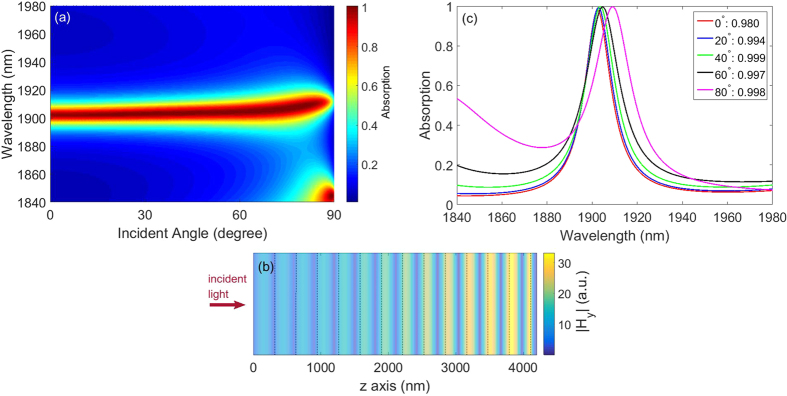
(**a**) The absorption spectra of the structure ((*CD*)_*S*_ *B*)_*N*_ on metal substrate for all angles of incidence. A flat and narrow-band absorption appears in the vicinity of 1902 nm. (**b**) The magnetic field distributions of the structure at 1902 nm for normal incidence. The dotted line indicates the interface between unit cell of 1DPC. (**c**) The absorption spectra of the structure at representative angles of incidence. The labels for the curves show the angles of incidence and the value of corresponding absorption peak.

When 1DPC is combined with metal substrate, TPPs exciting on 1DPC-metal interface give rise to strong light absorption. Using transfer matrix method, the absorption spectra of the structure are illustrated in Fig. 3(a). Intuitively, a flat and near-perfect absorption band appears in the vicinity of 1902 nm. Essentially, this near-perfect absorption band is resulted from TPPs existing on 1DPC-metal interface. As a typical example, we calculate the magnetic field distributions at 1902 nm for normal incidence in Fig. 3(b). It can be found that the Bloch wave in 1DPC along z axis is magnified exponentially and finally is localized on 1DPC-metal interface. The incident EM waves hence penetrate into metal substrate and are absorbed entirely by lossy metal. It should be pointed out that the narrow-band and wide-angle absorption is polarization-dependent. If we change TM polarization to TE, the wavevector dispersion of HMM is similar to an isotropic medium, and hence TPPs in the structure for TE polarization is dispersive which is similar to conventional TPPs. As a result, the narrow-band and wide-angle absorption can be obtained only for TM polarization but not for TE polarization.

In order to reveal the narrow-band and wide-angle absorption properties of TPPs clearly, we illustrate the absorption spectra of structure at representative angle of incidence in Fig. 3(c). On the one hand, the value of absorption peak is 0.98 for normal incidence. With an increase of *θ*, the value of absorption peak is always nearly 1, from 0.994 at 20 degree, to 0.998 at 80 degree. Therefore near perfect absorptions are exhibited for all angles of incidence. On the other hand, there is a slight wavelength shift with an increase of *θ*. The wavelength of absorption peak appears at 1902 nm for normal incidence, and at 1909 nm for 80 degree. The wavelength shift of absorption peak versus angle of incidence is just 7 nm. By contrast, the full width at half maximum (FWHM) of the absorption spectrum is 15 nm for normal incidence and 23 nm for 80 degree. FWHM of the absorption spectrum is much smaller than the wavelength of absorption peak, which exhibits a narrow-band absorption spectrum. Likewise, the wavelength shift of absorption peak is smaller than FWHM of the absorption spectrum, which exhibits wide angle near perfect absorption.

**Figure 4:**
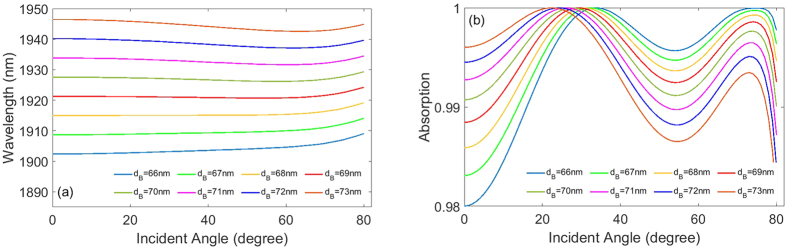
(**a**) The dependence of wavelength of absorption peak on angle of incidence at different thickness *d*_*B*_. (**b**) The dependence of value of absorption peak on angle of incidence at different thickness *d*_*B*_.

Moreover, our perfect absorber design is flexible for the structural parameters. The change of structural parameters does not essentially influence the performance of absorber. Here we change the thickness *d*_*B*_ and investigate the performance of absorption peak. Firstly, as mentioned above, the absorption peak shows a slight shift to long wavelengths (~7 nm) when *d*_*B*_ = 66 *nm*. If increasing the thickness *d*_*B*_, shown in Fig. 4(a), the wavelength shift of absorption peak becomes more slightly. When *d*_*B*_ = 69 *nm*, The wavelength of absorption peak even is invariant in the angle of incidence region of 0~70 degree, despite of slight shift to long wavelengths (~2 nm) in the angle of incidence region of 70~80 degree. Therefore, an optimized wide angle absorption behavior is exhibited in the case of *d*_*B*_ = 69 *nm*. If further increasing the thickness *d*_*B*_, the wavelength of absorption peak shifts to short wavelengths in the angle of incidence region of 0~70 degree, and then turns to long wavelengths in the angle of incidence region of 70~80 degree. However, the total wavelength shift of absorption peak is just 3 nm even if *d*_*B*_ increases to 73 nm. Anyway, the wavelength shift of absorption peak is still rather slightly. In addition, we can see from Fig. 4(b) that the value of absorption peak is always more than 0.98 in the angle of incidence region of 0~80 degree for the considered region of thickness *d*_*B*_.

## Conclusion

In summary, we have illustrated a simple design of a multilayer, wide-angle absorber exhibiting spectrally selective near-unity absorption. Spectrally selective perfect absorber is a specific application which requires both narrow band and wide-angle EM response. On the one hand, TPPs existing on 1DPC-metal interface induce penetration of EM waves into metal substrate, and then EM waves are absorbed entirely by lossy metal. On the other hand, phase variation compensation effect existing in 1DPC containing HMMs provide the methods of tailoring reflection phase, which can be used for realizing wide-angle TPPs under TM polarization. As a result, when 1DPC containing HMMs is combined with metal substrate, a narrow-band and wide-angle perfect absorption based on resonant tunneling effect is realized. The value of absorption peak is over 98% in the angle of incidence region of 0–80 degree and its FWHM just is 23 nm. Moreover, the simplicity of the structure is beneficial to its fabrication. We believe that such spectrally selective perfect absorbers possess potential applications for sensitive detectors and narrow-band thermophotovoltaic emitters.

## Additional Information

**How to cite this article**: Xue, C.-h *et al*. Wide-angle Spectrally Selective Perfect Absorber by Utilizing Dispersionless Tamm Plasmon Polaritons. *Sci. Rep.* **6**, 39418; doi: 10.1038/srep39418 (2016).

**Publisher's note:** Springer Nature remains neutral with regard to jurisdictional claims in published maps and institutional affiliations.
